# Supplementary figures and images for: A proteome-wide yeast degron collection for the dynamic study of protein function
Source: J Cell Biol. 2024 Dec 18;224(2):e202409050. doi: 10.1083/jcb.202409050 (PMC11654244; doi:10.1083/jcb.202409050)

Gpp1-AID-GFP

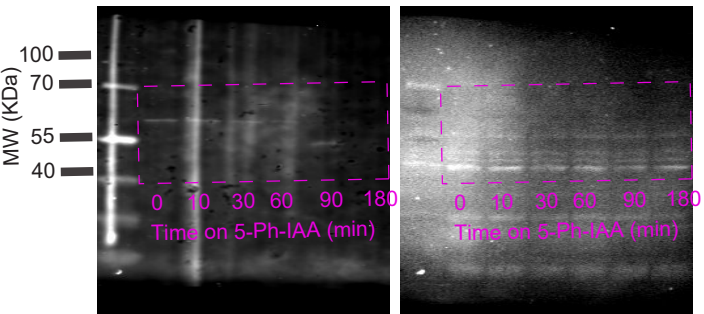

Tub3-AID-GFP

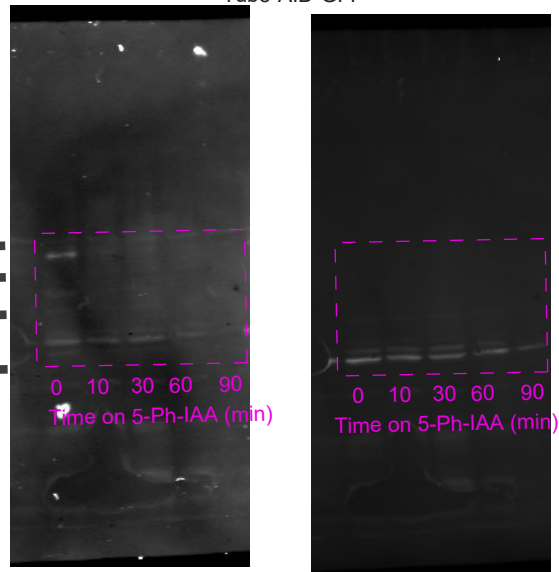

Npl3-AID-GFP

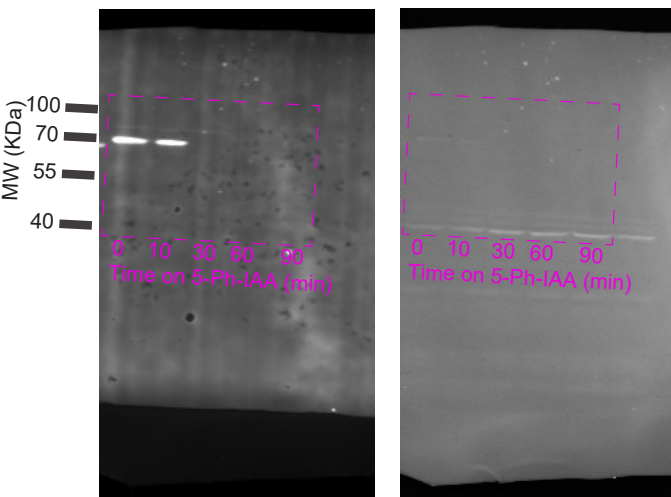

Supplement: SourceData F1 — is the source file for Fig. 1. [file jcb_202409050_sourcedataf1.pdf]
